# Development and evaluation of a robotic system for lumbar puncture and epidural steroid injection

**DOI:** 10.3389/fnbot.2023.1253761

**Published:** 2023-10-10

**Authors:** Jiaxin Lu, Zekai Huang, Baiyang Zhuang, Zhuoqi Cheng, Jing Guo, Haifang Lou

**Affiliations:** ^1^School of Automation, Guangdong University of Technology, Guangzhou, China; ^2^The Maersk Mc-Kinney Moller Institute, University of Southern Denmark, Odense, Denmark; ^3^The First Affiliated Hospital of Zhejiang Chinese Medical University (Zhejiang Provincial Hospital of Chinese Medicine), Hangzhou, China

**Keywords:** electrical impedance spectroscopy, surgical application instrumentation design, soft tissue, bio-impedance, Bayesian neural network, lumbar puncture, epidural steroid injection

## Abstract

**Introduction:**

Lumbar puncture is an important medical procedure for various diagnostics and therapies, but it can be hazardous due to individual variances in subcutaneous soft tissue, especially in the elderly and obese. Our research describes a novel robot-assisted puncture system that automatically controls and maintains the probe at the target tissue layer through a process of tissue recognition.

**Methods:**

The system comprises a robotic system and a master computer. The robotic system is constructed based on a probe consisting of a pair of concentric electrodes. From the probe, impedance spectroscopy measures bio-impedance signals and transforms them into spectra that are communicated to the master computer. The master computer uses a Bayesian neural network to classify the bio-impedance spectra as corresponding to different soft tissues. By feeding the bio-impedance spectra of unknown tissues into the Bayesian neural network, we can determine their categories. Based on the recognition results, the master computer controls the motion of the robotic system.

**Results:**

The proposed system is demonstrated on a realistic phantom made of ex vivo tissues to simulate the spinal environment. The findings indicate that the technology has the potential to increase the precision and security of lumbar punctures and associated procedures.

**Discussion:**

In addition to lumbar puncture, the robotic system is suitable for related puncture operations such as discography, radiofrequency ablation, facet joint injection, and epidural steroid injection, as long as the required tissue recognition features are available. These operations can only be carried out once the puncture needle and additional instruments reach the target tissue layer, despite their ensuing processes being distinct.

## 1. Introduction

Lumbar puncture (LP), sometimes called a spinal tap, is a critical step in accessing the spinal region and is irreplaceable in many medical diagnosis and treatment procedures, such as sampling spinal fluid for meningitis tests. The procedure has two main steps: the insertion of the spinal needle into the subarachnoid space filled with cerebrospinal fluid (CSF) and the subsequent therapeutic or diagnostic operations (Frederiks and Koehler, 1997). A study by Vickers et al. (2018) showed that ~363,000 LP procedures are performed in the USA every year.

**Figure 1:**
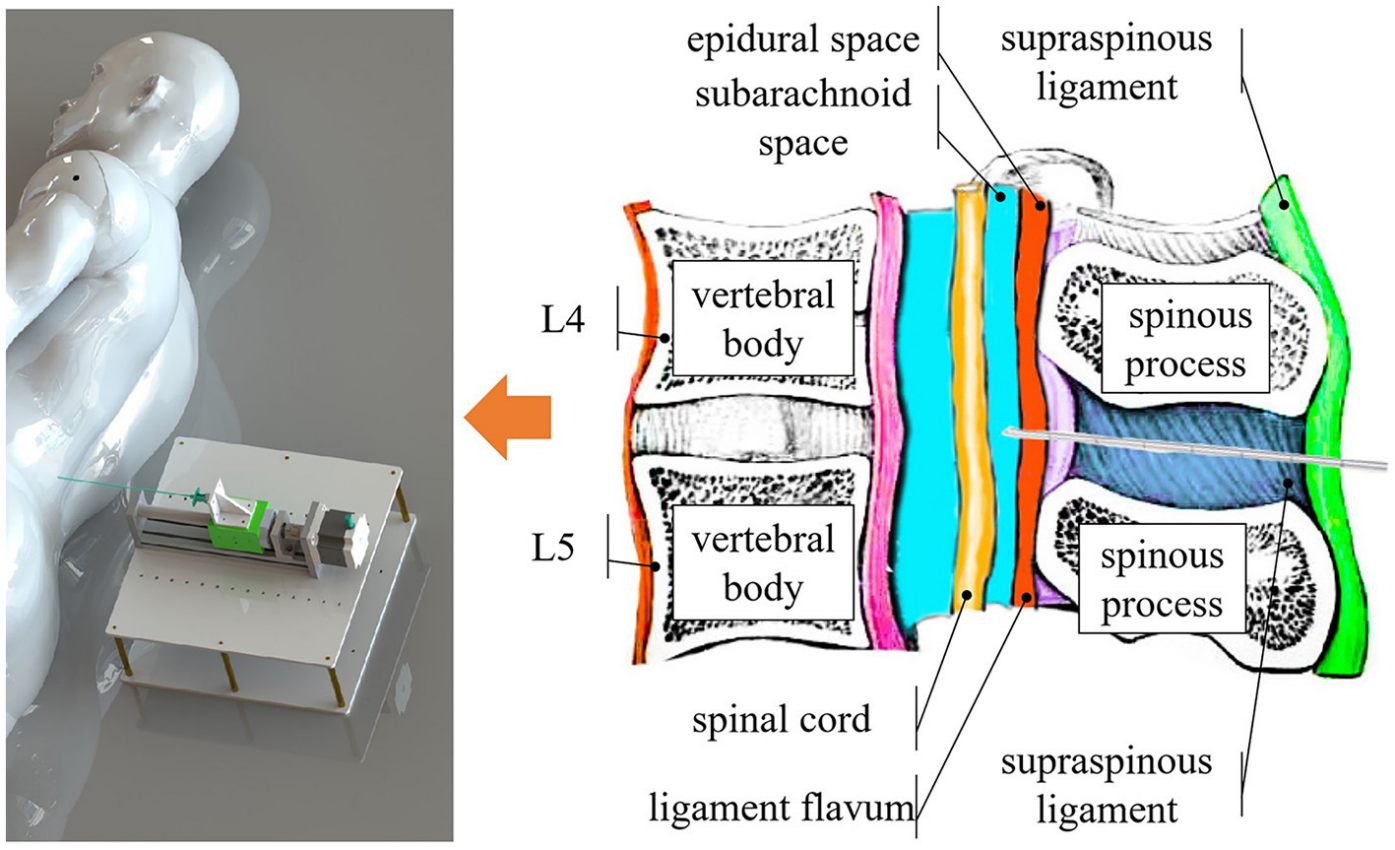
Overview of the system. Our system identifies different tissue layers during the puncture procedure by content, such as the epidural space, which is filled with fat, and the subarachnoid space, which contains CSF.

In a proper LP procedure, referring to Figure 1, the needle must be inserted into either the *L*_3_–*L*_4_ or *L*_4_–*L*_5_ interspinal spaces. The *L*_1_–*L*_2_ and *L*_2_–*L*_3_ interspinal spaces should be avoided due to the risk of injury to the conus medullaris (Roos, 2003). After penetrating the skin tissue, the needle passes through the supraspinous ligament, interspinal ligament, and ligamentum flavum. When the needle tip touches the dura mater outside the arachnoid membrane, the operator should feel a sudden resistance. Overcoming this resistance indicates that the needle tip has reached the subarachnoid space between the arachnoid and pia mater, where CSF can be sampled.

Although LPs are widely performed in clinical procedures, they do not always go smoothly. The individual physique of each patient is different, and obesity increases the risk of LP failure (Edwards et al., 2015). A physician's lack of experience may result in trauma at the perivertebral plexus of veins and blood vessels that accompany the latter in the subarachnoid space (Dripps and Vandam, 1951). In rare cases, the spinal cord may be injured by the needle if the procedure is performed at an improper level (Evans, 1998). Overall, there is potential for patients to suffer from various traumas in the LP procedure. Doctors could be confused about whether blood in the CSF is pathological or has been caused by extra trauma (Solomon, 1935).

Several methods have been developed to ensure the accurate insertion of the puncture needle into the subarachnoid space where CSF is located to avoid unnecessary injuries. One common method is fluoroscopically guided LP, which uses X-ray imaging to guide the needle. However, this method has some drawbacks, such as ionizing radiation (Cauley, 2015) and side effects from the contrast agent (Samkoe et al., 2018). Therefore, fluoroscopy guidance is often used as a backup option for failed LPs, requiring an extra procedure (Edwards et al., 2015). Another method is ultrasound-guided LP, which uses sound waves to visualize the soft tissues (Pierce et al., 2018). This method has the advantages of being non-radiative and noninvasive. However, both methods require bulky and costly equipment, which limits their availability in remote and low-resource settings.

As a low-cost and portable method, electrical bio-impedance (EBI) technology has manifested its value in different applications. EBI technology is a cluster of methods which aims to analyze EBI data from biological tissues. By fitting the Fricke and Morse (1925)'s bio-electricity model, Guermazi et al. (2014) quantify the composition of biological tissue. In eye surgery for puncture detection, Schoevaerdts et al. (2019) integrate electrodes in a puncture needle to monitor the bio-impedance variance. Halter and Kim (2014) generate electrical bio-impedance tomography for abnormal tissue detection. And Van Assche et al. (2023) use EBI as proximity sensing in neuroscience research.

We refer to some related studies using various approaches to detect the subarachnoid space. Several methods use tactile devices that monitor the force feedback and its change or derivative as the needle tip penetrates through different soft tissue layers (Singh et al., 1994; Ambastha et al., 2016; Li et al., 2021; Wang and Li, 2022). A different CSF detection method proposed by Sievänen et al. (2021) uses a bio-impedance needle, which measures the electrical impedance of the tissues as a function of time by applying and sensing an alternating current. Both methods have shown feasibility for use in LP.

Unlike Sievänen's CSF detection system, our work focuses on robotic engineering and tissue identification. A clear advantage of the robotic system is that it can stop at any specified target tissue layer. Another advantage is that the robot is highly stable when controlled to stop and remain in place. Therefore, this system has the potential to automate various procedures besides LP, such as epidural steroid injection (ESI), radiofrequency ablation (RFA), and discography. When applied to ESI, users only need to detect the epidural fat layer. As for RFA and discography, the needle can stop at the corresponding position.

In our research, we developed a robotic system with one degree-of-freedom that uses a bio-impedance needle as the sensing unit. Instead of finding a proper frequency, our system acquires the whole impedance spectrum as the feature of the tissues. We developed a method for classifying and identifying soft tissue layers based on sampling bio-impedance spectrum data from 1 to 349 kHz and applying a Bayesian neural network (BNN) to the sampling data. Halonen et al. (2019) has proved that vivo tissues can be accurately identified by bio-impedance needles using bio-impedance spectrum scanning technology. Another investigation by Denkçeken et al. (2020) implied that the bio-impedance spectrum is sufficiently reliable and precise to reveal the presence of different bacteria in CSF, even under the challenging condition of CSF samples with similar electrical properties. However, our work focused on the identification algorithm and application in robot-assisted LP. Our design combines the robotic system with a master computer. We further investigated the characteristics of the system through a series of phantom experiments.

To avoid traumatic puncture, the needle tip should not travel too far into the subarachnoid space, which is a very thin ring-shaped layer inside the spinal column. We define the comfortable travel distance (CTD) as the distance from the first contact of the needle tip with the CSF and its final stopping point. According to Holsheimef and Barolat (1998), the minimum width of the dorsal CSF layer in 106 patients ranges from 2 to 3 mm. Therefore, we set our CTD to be <2 mm, taking into account engineering redundancy.

The remainder of this paper is organized as follows. Section 2 describes the robotic system in detail and tests its performance. Section 3 illustrates how the BNN is implemented on the master computer. Section 4 presents the results of phantom experiments for the preliminary verification of the tissue-identifying system for robot-assisted LP by operating and recording the needle penetration process. Finally, Section 5 concludes the paper with a discussion of the test results and expectations for future applications and development.

## 2. Robotic system

Robot-assisted surgery has developed rapidly since the first platform was used in 1985 (Morrell et al., 2021). Robot assistance offers surgeons greater efficiency and stability. In this paper, we present a robotic system that uses a bio-impedance needle to identify different tissue layers and stop at the desired depth. As shown in **Figure 3**, the system consists of a bio-impedance needle, an impedance spectrum analyzer, a closed-loop stepper motor, and a master control panel. Our design improves the accuracy of LP by stopping the needle as soon as it reaches the subarachnoid space.

### 2.1. Needle probe design

**Figure 2:**
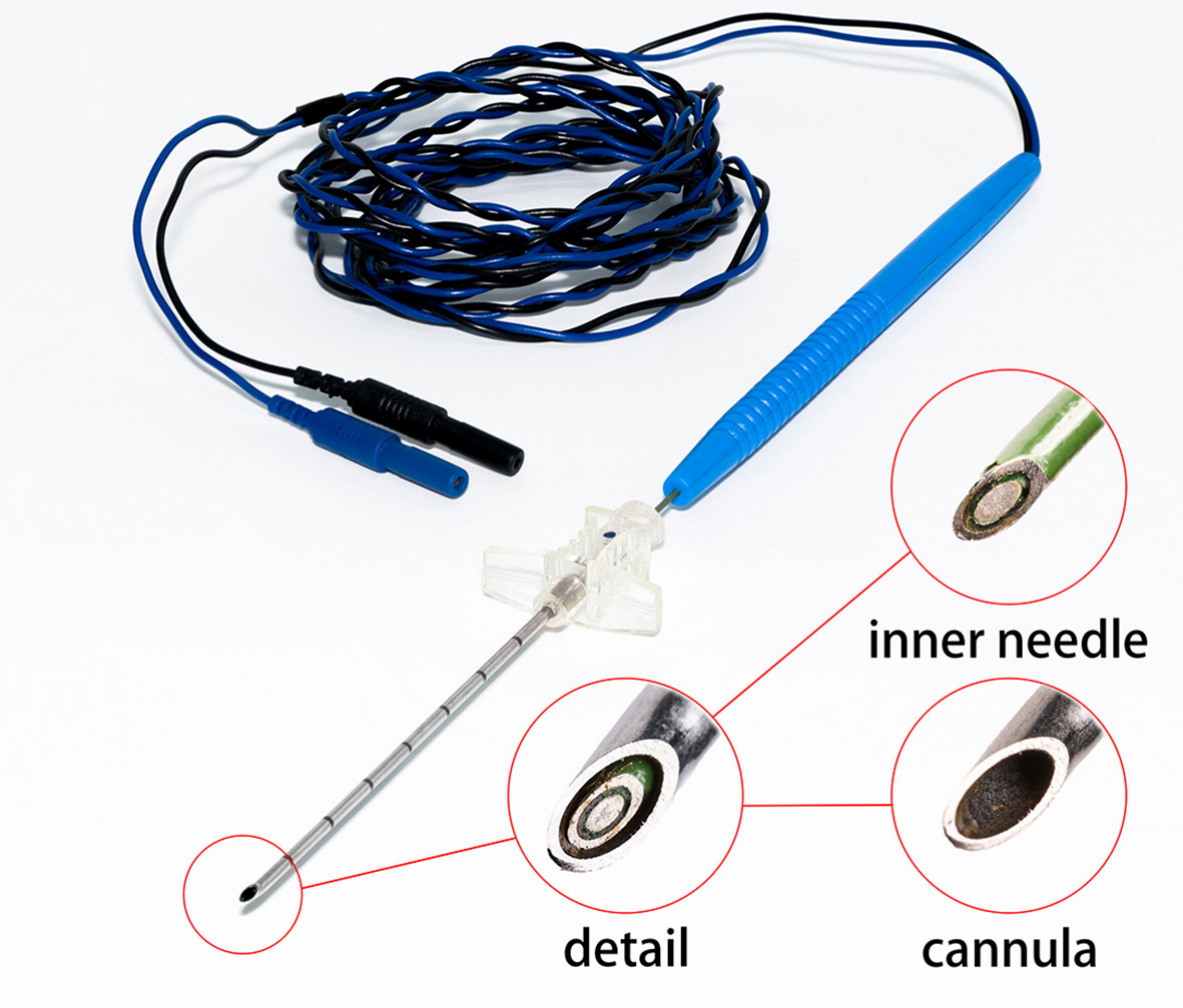
Bio-impedance needle. The diameter of the inner needle is 0.90*mm*. The external diameter of the cannula is 1.60*mm* and the internal diameter is 1.10*mm*.

Inspired by Cheng et al. (2016, 2019), the bio-impedance needle for LP is formed of a stainless-steel cannula and an inner needle with two electrodes. The inner needle is a concentric electrode, as shown in Figure 2. An insulator separates the electrodes and covers most of the outer electrode's surface. This prevents the outer electrode from touching the metal cannula. The cannula protects the inner needle from the tough ligament during LP. The sensor is a two-wires configuration and only the inner needle connects to the impedance spectrum analyzer.

### 2.2. Robotic system structure

**Figure 3:**
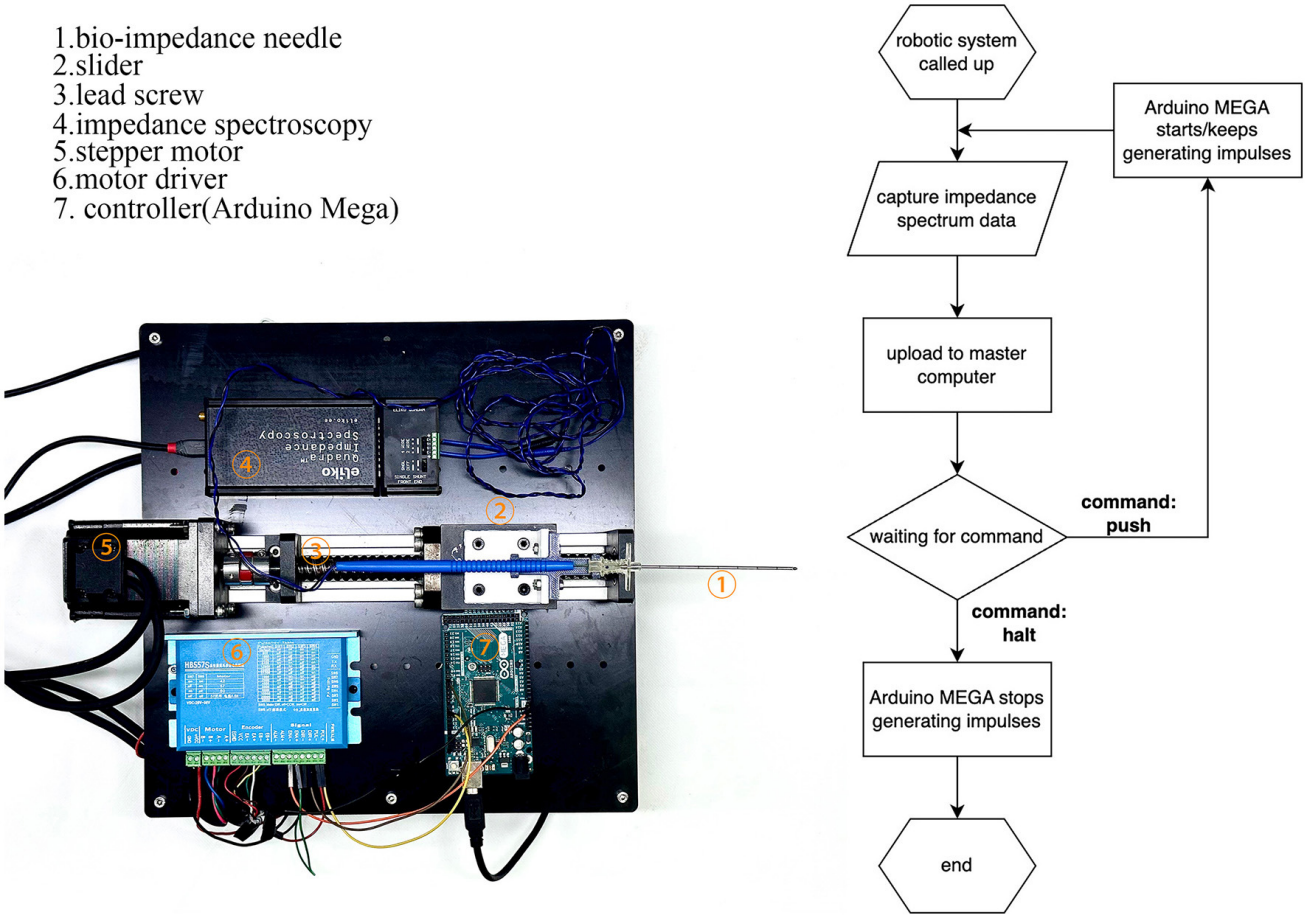
Construction of the robotic system and flow diagram of its operation. The robotic system feeds bio-impedance data to the master computer and moves according to the master computer's identification from the data.

The robotic system, shown in Figure 3, consists of a puncture needle, stepper motor, motor driver, Arduino MEGA board (Arduino Inc., Italy), impedance spectrum analyzer, and mechanical structures. The stepper motor drives the lead screw, which moves the slider horizontally on the slide track. This limits the bio-impedance needle's movement to one degree-of-freedom. The Arduino MEGA board generates an impulse current to control the stepper motor. The impedance spectrum analyzer (Quadra Impedance Spectroscopy, designed by Eliko.cc, Estonia) measures the impedance spectrum from the bio-impedance needle and sends data to the master computer when commanded. The robotic system follows the process illustrated by the flow diagram in Figure 3.

Once the cannula is in position, users can replace the inner needle with other surgical devices for sampling, anesthesia, or curing.

### 2.3. Robotic performance

**Figure 4:**
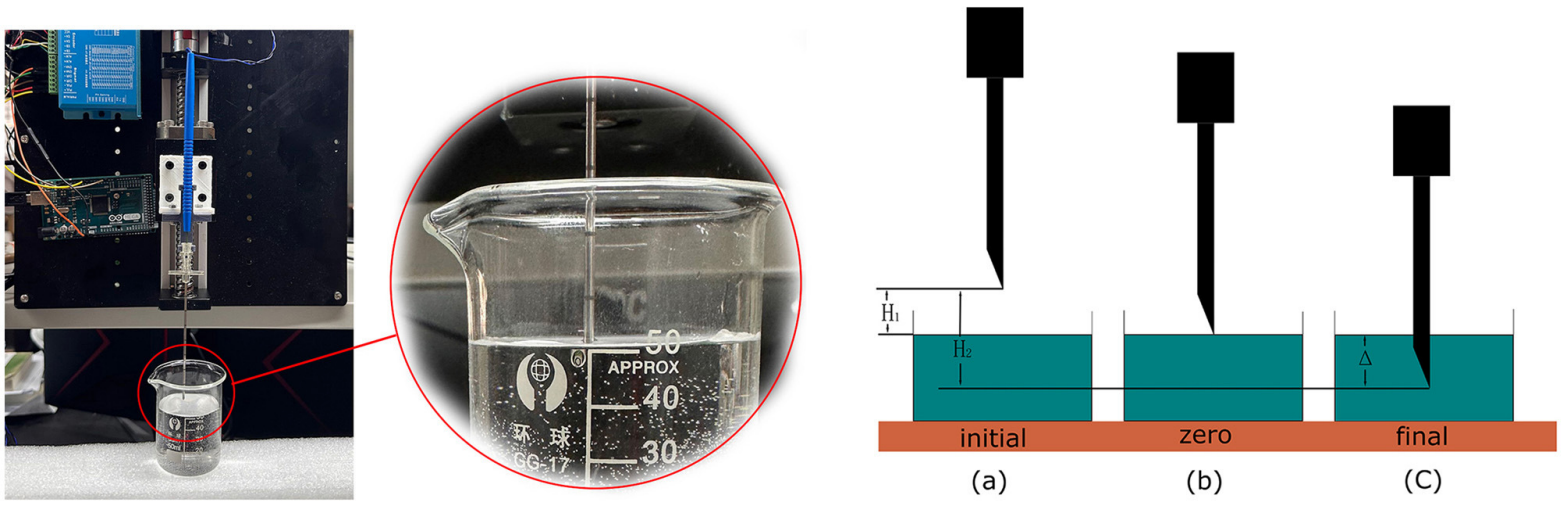
Performance test of the robotic system to measure the CTD. We set up three marked positions to measure the CTD.

To verify the robotic performance, we measured the CTD Δ in Figure 4, which is the distance between the first contact of the needle tip with the saline solution and its final stop. We mounted the robotic system vertically, as shown in Figure 4, and drove the bio-impedance needle from the air into a container of saline solution. We used an Arduino program to record the depth of the needle movement by counting the impulses of the stepper motor. The counter increases by 1 for each forward impulse and decreases by 1 for each backward impulse.

The detection process started with two preparation steps. In step 1, we set the needle at a specified height above the liquid surface, as shown in Figure 4(a), marked as the initial position, and reset the counter to 0. In step 2, we manually controlled the stepper motor to lower the needle to the liquid surface. We adjusted the needle carefully by turning the stepper motor clockwise or anticlockwise until the needle tip just touched the liquid surface, as shown in Figure 4(c). We marked this as the zero position and recorded its value as *N*_1_. The depth from the initial position to the zero position is *H*_1_. Taking *H*_*s*_ to be the distance that the stepper motor moves in a single step, we have


(1) $$\begin{array}{l} H_{1}=N_{1}H_{s} \end{array}$$

Using a homing function in Arduino, we drove the needle back to the initial position by a command from the master control panel. We then began the detection process. Similar to the first experiment, the needle stopped when the master control panel detected CSF. We marked this position as the terminal position *N*_2_ in Figure 4(c). The depth from the initial position to the terminal position is *H*_2_. Thus, we have


(2) $$\begin{array}{l} H_{2}=N_{2}H_{s} \end{array}$$

Then,


(3) $$\begin{array}{l} \begin{array}{c} \Delta =H_{2}-H_{1}\\ =\left(N_{2}-N_{1}\right)H_{s} \end{array} \end{array}$$

where Δ is the difference between *H*_1_ and *H*_2_, which reflects the detection distance of this robotic system. The value of Δ depends on *H*_*s*_, which can be adjusted by the motor driver from 0.08 to 0.22 mm, and the difference between *N*_1_ and *N*_2_, which challenges the system's performance. After 20 repeated experiments, the mean difference between *N*_1_ and *N*_2_ was found to be 21.65 and STD is 1.93. As we configured the stepper motor to the most accurate mode, the CTD Δ turns out to be 1.732 ± 0.154*mm*, satisfying the requirement for a maximum of 2*mm*.

## 3. Programs on the master computer

This section discusses the GUI and functions implemented on the master computer. The master computer's tasks mainly concern receiving data from the robotic system, data processing, classifying the tissue type, and sending commands to the robotic system.

First, the user should set a target on the GUI. Having started the robotic system, the master computer continually receives data. The uploaded data are processed in the Data Remapping Function and then sent to the BNN classifier. The output from the BNN is the current tissue layer. Once the target tissue layer has been identified, the master computer sends a stop command to the robotic system so that the bio-impedance needle halts at the right position. The main challenge for the master computer is to correctly identify CSF from the bio-impedance spectrum.

BNNs effectively solve the overfitting problem, especially in the case of small datasets. In a standard artificial neural network, the dataset should be divided into a training set and a test set, where the test set should be large enough to reduce the signal-to-noise ratio in the test error. However, BNNs do not need to sacrifice part of the data to the test set (MacKay, 1992b) because the Bayesian evidence provides a reference to validate. Another problem of standard artificial neural networks is overfitting. For BNNs, overfitting is difficult because they are mainly trained using the necessary weights in the network (Burden and Winkler, 2009), while the unnecessary weights converge to zero during the training procedure.

Different to standard neural networks, BNNs treat the weight as a random variable instead of an assigned value. The initial weights (or priors) are usually defined as a standard normal distribution. Therefore, the trained parameters are the mean and standard deviation of the normal distribution. Instead of maximum likelihood estimation, the learning process identifies the maximum a posteriori of the probability of random weights (MacKay, 1992a).

Serving as a classifier, the output units from the BNN are also random. However, we choose the maximum from the means of the output units to determine the classification result. The error corresponding to the maximum mean value determines the confidence level. For a classifier, a popular cost function for the back-propagation method is the cross-entropy of the dataset.

### 3.1. Graphical user interface

**Figure 5:**
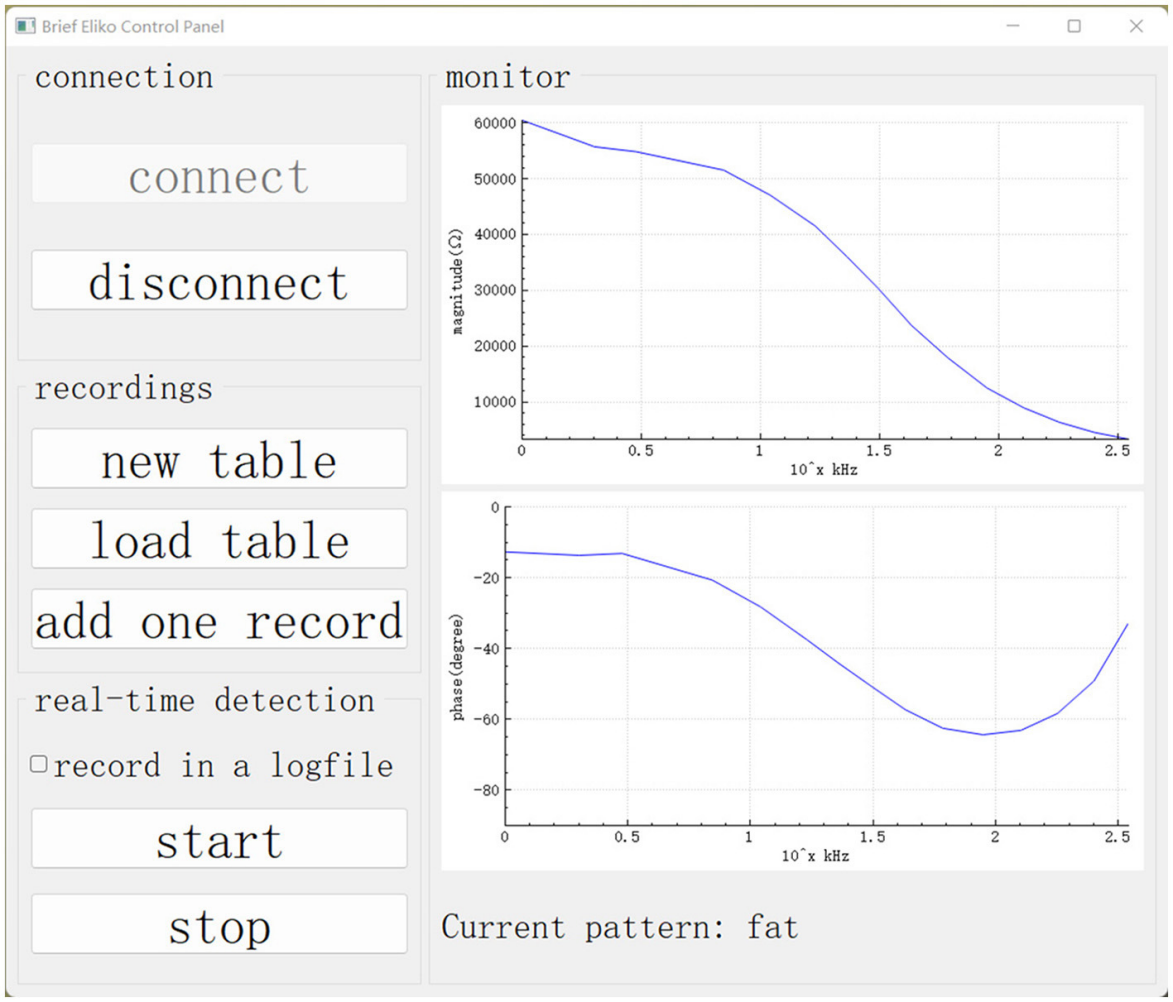
GUI on the master computer. Our data collection and real-time detection are called up using this GUI.

The GUI in the master computer is based on the Qt framework and plays the role of control panel and monitor. As shown in Figure 5, the buttons on the left side are designed to control functions running in our experiment. On the right side, there is a monitor displaying the impedance spectrum in the form of a bode plot. The monitor allows the user to visually check abnormal results and manually stop the robotic system.

### 3.2. Feature remapping function

To generate a BNN classifier, we must first build a dataset. The dataset includes samples with labels and features. In the dataset, the samples are recorded by rows while their labels and features are arranged in columns.

Impedance spectroscopy captures the bio-impedance of the tissues, but it is the conductivity of the tissues that leads to different bio-impedance. Moreover, the captured bio-impedance data are influenced by different concentric needle electrodes and their manufacturing tolerances. To calibrate the differences introduced by such deviations, our previous research (Cheng et al., 2020) developed a calibration method whereby the captured impedance values |*Z*| are remapped to the conductivity σ. The remapping function of a concentric electrode is generated by measuring the impedance of a saline solution of known concentration |*Z*_*S*_|, because conductivity σ_*S*_ is a dielectric constant of saline solutions that only varies according to concentration.

In other words, the remapping function involves the extraction of the correlation between |*Z*_*S*_| and σ_*S*_. As vivo tissues share the same correlation as saline solution, the *Z* value of the target tissue can be remapped to its conductivity σ through the same remapping function.

**Figure 6:**
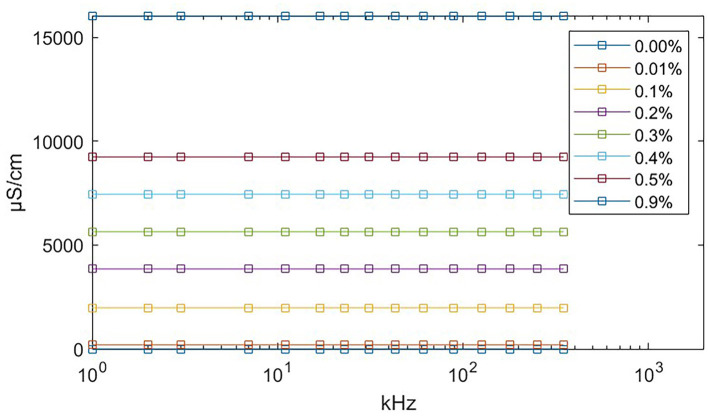
Theoretical conductivity values of saline solution relating to excitation current frequency. The theoretical value remains constant at all frequencies. The exact conductivity value is given in Table 1.

We used eight salinecenter solutions with different concentrations (0.00, 0.01, 0.1, 0.2, 0.3, 0.4, 0.5, and 0.9%, where 0.00% salinity corresponds to distilled water with a conductivity of 5 μ*S*/*cm*) to cover the impedance range of the target tissues. Figure 6 shows their theoretical values (Stogryn, 1971) as a function of frequency. We measured the saline solutions and generated a remapping function for our device based on Table 1, which lists the conductivity values and captured impedance values. The conductivity values are given in microsiemens per centimeter (μ*S*/*cm*) and are independent of the excitation current frequency. The impedance values are measured in ohm (Ω) and vary with frequency. Each impedance value is the median of 500 samples from the corresponding saline solution.

**Table 1:** Remapping table.

**FREQ**	**Concentration of saline solution**							
	**0.00%**	**0.01%**	**0.1%**	**0.2%**	**0.3%**	**0.4%**	**0.5%**	**0.9%**
COND	5	200	1,987	3,850	5,650	7,450	9,238	16,000
1 kHz	305,009	30,744	8,657	6,607	5,547	4,885	4,453	3,856
2 kHz	238,883	27,665	7,087	5,198	4,257	3,619	3,260	2,709
3 kHz	210,094	26,599	6,599	4,776	3,891	3,278	2,939	2,404
7 kHz	119,218	23,260	5,395	3,755	2,970	2,408	2,121	1,638
11 kHz	83,554	21,773	4,959	3,403	2,658	2,120	1,853	1,389
17 kHz	55,698	19,764	4,589	3,118	2,417	1,902	1,651	1,204
23 kHz	42,127	17,997	4,340	2,941	2,270	1,776	1,537	1,105
31 kHz	31,820	16,183	4,100	2,775	2,135	1,658	1,432	1,015
43 kHz	23,303	14,031	3,840	2,604	2,000	1,543	1,332	929
61 kHz	16,604	11,576	3,550	2,423	1,863	1,431	1,236	851
89 kHz	11,498	8,897	3,207	2,215	1,707	1,305	1,130	766
127 kHz	8,066	6,634	2,845	2,005	1,555	1,188	1,033	692
179 kHz	5,706	4,889	2,461	1,784	1,401	1,075	939	623
251 kHz	4,055	3,563	2,077	1,558	1,242	961	844	557
349 kHz	2,939	2,630	1,734	1,349	1,097	859	759	503

The feature remapping function works as follows. From Table 1, we find that the conductivity σ_*S*_ increases and the impedance |*Z*_*S*_| decreases with increasing concentration at each frequency. Therefore, if we plot the impedance along the x-axis and the conductivity on the y-axis, we obtain a monotonically decreasing function for each frequency. When the master computer receives the bio-impedance spectrum data from impedance spectroscopy, it linearly interpolates the bio-impedance values into the saline impedance values at their corresponding frequency and obtains their conductivity.

To summarize, the remapping function converts the impedance spectrum |*Z*| to the conductivity spectrum σ using linear interpolation according to Table 1. For the soft tissue dataset and the BNN classifier, we use the conductivity spectra from 1 to 349 kHz as 15 features.

### 3.3. Dataset preview

**Figure 7:**
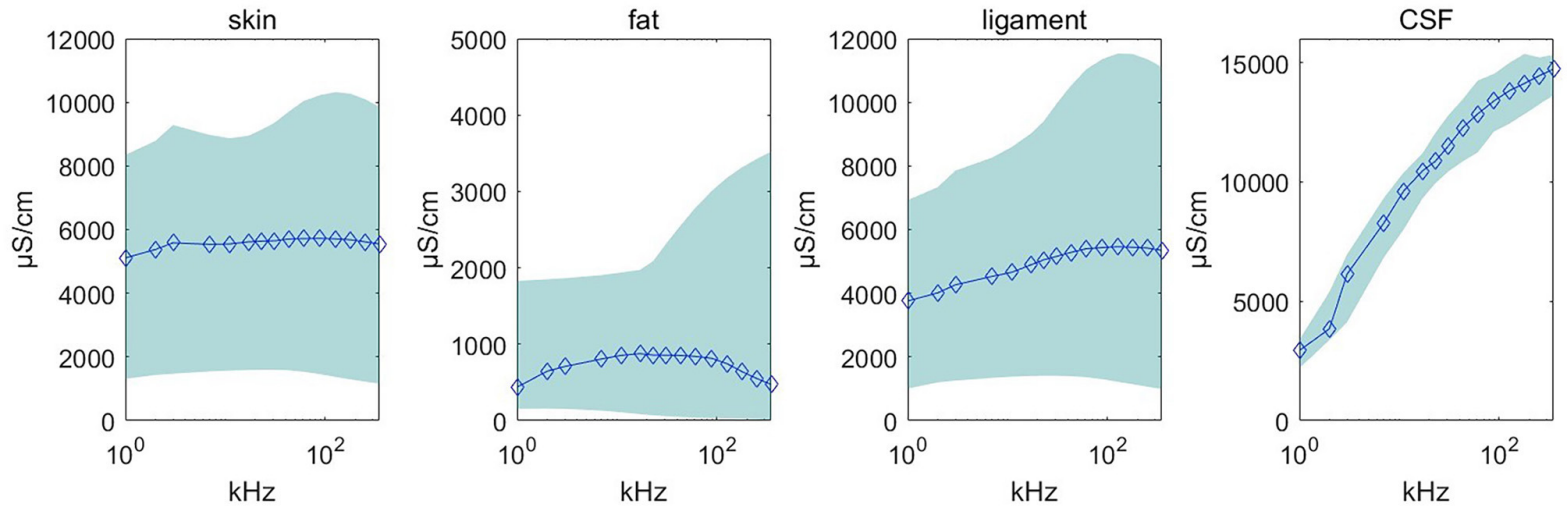
Conductivity spectra of the tissues and CSF. This chart records the conductivity remapped from impedance over frequencies from 1 to 349 kHz for all samples in Table 2. The rhombus is the median value at one of the 15 measured frequencies. The pale blue error bar is the range of the samples.

Using the feature remapping function, our device is able to identify the target tissues of skin, fat, ligament, and CSF. These soft tissues can be sampled from a pig's foot. The soft tissue dataset presented in Table 2 was formed using data from eight pig feet. Figure 7 visualizes the soft tissue dataset, showing the conductivity spectra of the samples in terms of their range and median values.

**Table 2:** Soft tissue dataset.

**No**.	**Tissue name**	**No. of samples**
1	Skin	150
2	Fat	81
3	Ligament	149
4	CSF	146

### 3.4. Design and fitting of BNN

This section describes the preparation, design, and fitting of the BNN classifier. the BNN was developed in Python 3.10 using PyTorch (version 1.13.1), torchbnn (version 1.2), an Nvidia 3070ti GPU, and CUDA11.7 (GPU toolkit). Thus, the BNN was trained using a GPU. The fitting process is described in the following steps:

1. We set up a BNN with two hidden layers, and set the weight priors to obey a standard normal distribution using the torchbnn framework. This neural network has one input layer with 15 units, one output layer with four units, and two hidden layers. Each of the hidden layers contains 10 units. Under a Bayesian approach with a forward function, the weights are assumed to be normally distributed, and all weights are set to *mean* = 0 and *std* = 1 as the prior distribution. As a classification task, the output is a one-hot encoding categorical variable marked by units in the output layer, so there are four units corresponding to the four targets.

2. The loss function is regularized by the Kullback–Leibler divergence (KLD), also called the relative entropy, from the weights. A popular way of regularizing the loss function is to add the KLD multiplied by a coefficient of influence to the cross-entropy (CE):


$$cost=CE+\frac{1}{batch\text{\_}size}KLD,$$

where *batch*_*size* is the number of samples in a training batch. These functions are integrated in the torchbnn framework. As the dataset is small, we take the whole dataset as a single batch. Thus, the batch size is the number of samples.

**Figure 8:**
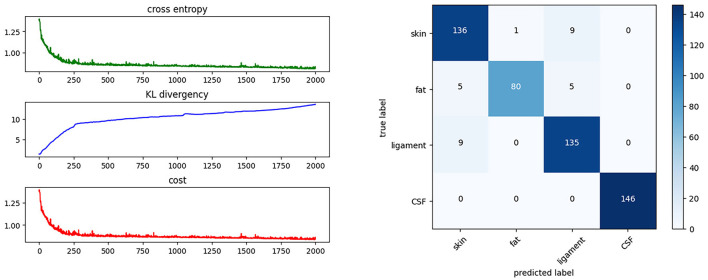
Training progress and results. This shows the cost function during the training process and the confusion matrix of the BNN classifier.

3. The fitting process is iterated over 2,000 epochs using the back-propagation method, as shown in Figure 8. The cost function decreases on each iteration and converges to a constant. Figure 8 shows the cost after each iteration. The fitting result can be described using a confusion matrix, as shown in Figure 8. The confusion matrix reveals the misclassification relationship between the tissues.

## 4. Preliminary phantom experiments

This section describes the results of phantom experiments performed to check the performance of the robotic system and the BNN classifier in a simulation environment.

### 4.1. Lumbar puncture phantom

**Figure 9:**
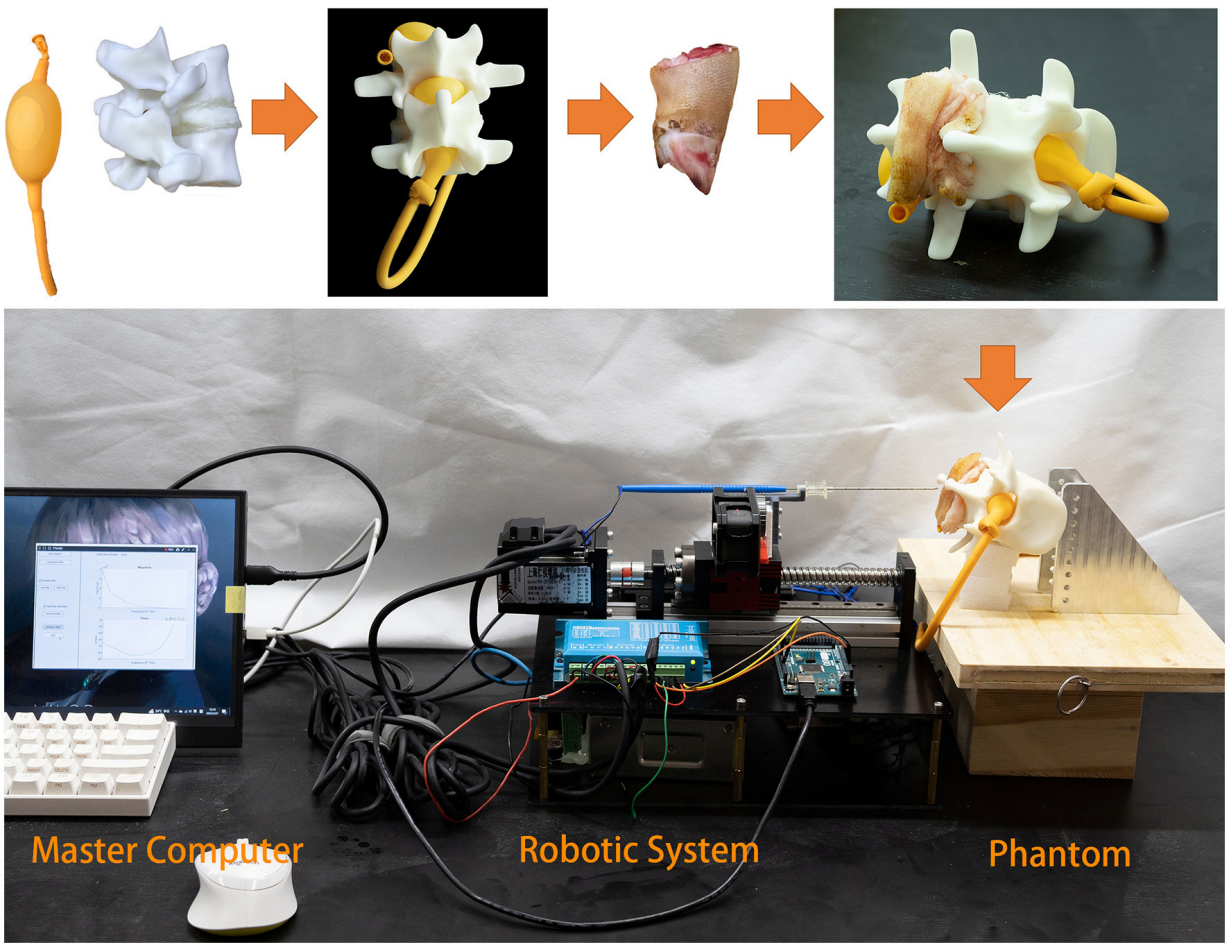
Phantom experiment. The balloon is used to simulate the inter-spinal space. As the balloon is elastic, it fits inside the lumbar spinal model. Fat and ligament from a pig's foot are placed on the spinal model.

As shown in Figure 9, we created a simulation environment for LP and ESI using a pig's foot, a spinal model, and a balloon filled with 0.9% saline solution. The pig's foot consists of soft tissues and bone, but we collected the skin, fat, and ligament to simulate the human soft tissues. The balloon represents the subarachnoid space that contains CSF. We used saline solution instead of real CSF because, with a high-frequency excitation current, the conductivity of CSF is close to that of 0.9% saline solution, namely 16,000 μ*S*/*cm*.

### 4.2. Experiment I

**Figure 10:**
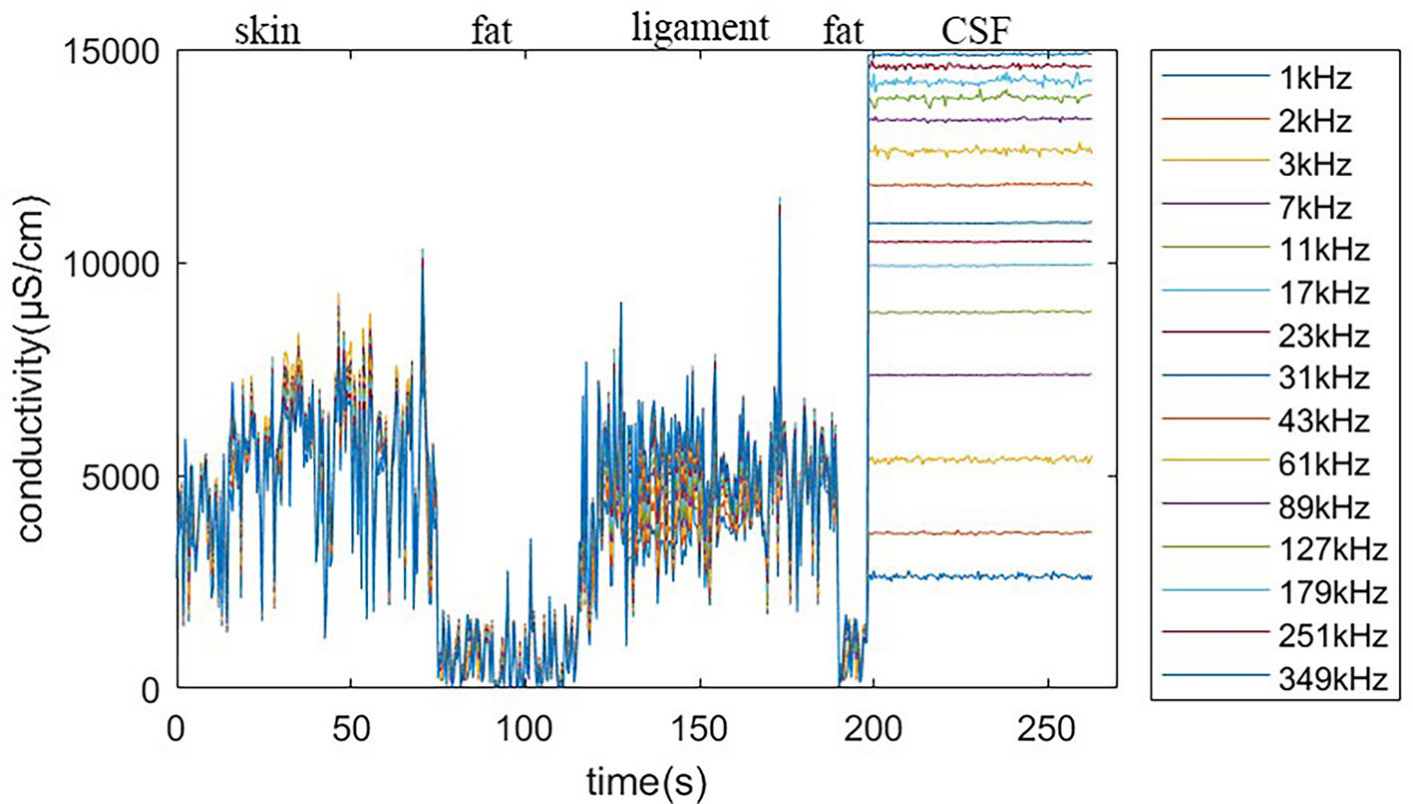
Real-time detection log.

In experiment I, we simulated part of the LP procedure. The target is CSF, so the needle did not stop moving forward until it arrived inside the balloon. The operator could start the procedure and check the needle tip position in real time through the graphical bio-impedance spectrum and textual real-time results on the monitor of the master computer. The master computer also generated a log file of the detection data and textual results during the experiment. The detection data and results are visually presented in Figure 10.

**Figure 11:**
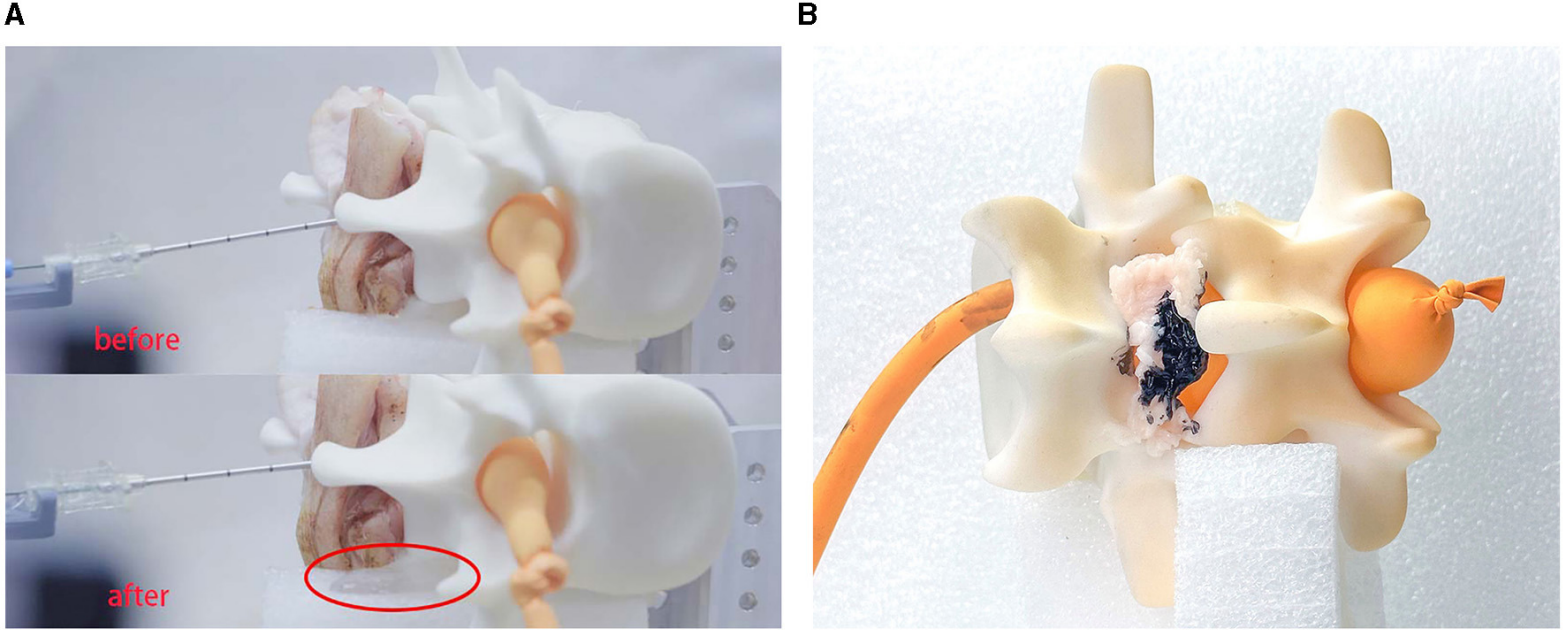
Experimental results. This experiment verified the feasibility of the robot system for medical procedures. **(A)** Shows the results for LP and **(B)** shows the results for ESI.

It can be seen that the saline solution has leaked out in Figure 11A, at which point the classification result shows “CSF” and the needle has stopped. Over 10 repeated experiments, our system was able to penetrate the soft tissues and then stop precisely at the target within the expected CTD. Two of the 10 experiments failed because of needle bias; the other eight experiments all succeeded. This indicate that the cannula structure of the needle is not sufficiently robust for real surgery.

### 4.3. Experiment II

In experiment II, we simulated the puncture procedure of ESI. In this experiment, the balloon should not be impaled, the needle tip should stop at the epidural fat layer and the final position of the needle tip is marked by ink. We set fat as the target tissue and introduced a time delay for the tissue-identifying program to avoid detecting subcutaneous fat. Once the needle had stopped, we injected a small drop of ink into the cannula to mark the tissue. We then disassembled the phantom environment to check the location of the ink, as shown in Figure 11B.

## 5. Conclusion and discussion

This study has presented a novel tissue identification system for robot-assisted LP. The system can distinguish different tissues during LP procedures by measuring the bio-impedance spectrum at the needle tip. This multi-classification method provides more information to the users, as they know exactly which tissue layer the needle tip has reached in real time. We performed preliminary experiments simulating LP and ESI on a realistic phantom made of *ex-vivo* animal tissue, and showed that the system was feasible and effective for tissue identification.

One problem encountered in the preliminary experiment was the deformation of the bio-impedance needle. In further research, a stronger structure should be considered for the junction between the cannula and the slider. The robotic performance of this system is also limited in the absence of robotic motion compensation for respiratory movement.

Further developments will include improvements to the needle probe design, refinement of the mechanics, and a more persuasive experimental environment. Regarding the needle probe, a refined electrode design of the inner needle would contain three or four electrodes so that the voltage signal could be sampled without any excited current interference in the measurements (Grimnes and Martinsen, 2015). Regarding the mechanics, further research will focus on a faster and more precise structure. Simulations of the bio-electric interference in living bodies would make the experiments more realistic.

## Data availability statement

The original contributions presented in the study are included in the article/Supplementary material, further inquiries can be directed to the corresponding authors.

## Ethics statement

The studies involving human participants were reviewed and approved by the Ethic Committee of Guangdong University of Technology. Written informed consent to participate in this study was provided by the participants. This study also uses animal samples obtained from public source. The Ethic Committee of Guangdong University of Technology did not require the study to be reviewed or approved by an ethics committee because the source of the animal samples follows the the local law.

## Author contributions

JL: Conceptualization, Data curation, Investigation, Software, Writing—original draft, Writing—review and editing. ZH: Investigation, Validation, Writing—review and editing. BZ: Investigation, Writing—review and editing. ZC: Conceptualization, Formal analysis, Methodology, Supervision, Writing—review and editing. JG: Conceptualization, Funding acquisition, Methodology, Project administration, Resources, Supervision, Writing—review and editing. HL: Writing—review and editing.
